# Somatic Embryogenesis and Plantlet Regeneration in the *Carica papaya* L. cv. Eksotika

**DOI:** 10.3390/plants9030360

**Published:** 2020-03-12

**Authors:** Baker Al-Shara, Rosna Mat Taha, Jamaludin Mohamad, Hashimah Elias, Asif Khan

**Affiliations:** 1 Institute of Biological Sciences, Faculty of Science, University Malaya, 50603 Kuala Lumpur, Malaysia; rosna@um.edu.my (R.M.T.); jamal@um.edu.my (J.M.); asif.khan.qau@siswa.um.edu.my (A.K.); 2 Faculty of Resource Science and Technology, University Malaysia Sarawak (UNIMAS), 94300 Kota Samarahan, Sarawak, Malaysia; ehashimah@unimas.my

**Keywords:** activated charcoal, indole-3-butyric acid, papaya, phloroglucinol, polyethylene glycol, somatic embryogenesis

## Abstract

A highly efficient protocol for regeneration of *Carica papaya* L. cv. Eksotika somatic embryos from immature zygotic embryos was developed. This study was designed to overcome the obstacles in regeneration of somatic embryos from immature zygotic embryos of “Eksotika”, especially problems associated with formation of better root quality and callus formation at the base of somatic embryos. Somatic embryos were generated by incubation of immature zygotic embryos in half-strength salt Murashige and Skoog (MS) medium with full-strength vitamins supplemented with 7.5 mg L^−1^ 2,4-D, 100 mg L^−1^ L-glutamine, 50 mg L^−1^ *myo*-inositol, 45 mg L^−1^ adenine sulphate, 0.33% gelrite, and 6% sucrose, followed by transfer to maturation medium consisting of ½ MS medium supplemented with 5 mg L^−1^ phloroglucinol, 100 mg L^−1^ L-glutamine, 100 mg L^−1^ *myo*-inositol, 68 mg L^−1^ adenine sulphate, 0.38% gelrite, and 3% sucrose. After that, well-formed somatic embryos were transferred to MS medium containing 3% sucrose and 0.8% agar for shoot production. The embryos were elongated in MS medium supplemented with 1 mg L^−1^ gibberellic acid, 0.5 mg L^−1^ indole-3-butyric acid, 100 mg L^−1^ *myo*-inositol, and 3.76 mg L^−1^ riboflavin. Root regeneration was achieved on MS medium containing 7.9 mg L^−1^ phloroglucinol and supported with vermiculite after 4 days of cultivation on ½ MS medium with 2 mg L^−1^ indole-3-butyric acid. After the rooting phase, in vitro plantlets were acclimatized in peat moss soil.

## 1. Introduction

*Carica papaya* (Caricaceae, Papaya), especially the “Eksotika” cultivar, is a significant crop in Malaysia [1]. *C. papaya* is considered as an economically important fruit tree, especially for tropical and subtropical populations. The *C. papaya* fruit can either be consumed as a fresh fruit or used as a treated product. The unripe fruits contain latex, which is the source of papain, a plant proteolytic enzyme [2]. The papain enzyme is able to break down protein and polypeptide. Therefore, it plays a crucial role in drug purposes and pharmaceutical industry. It is also used for clearing beer, tenderizing meat, leather industry, cosmetics industry, and candy and chewing gum industry [3,4].

Improvement of *C. papaya* could be achieved by increasing yield and improving the qualitative characteristics of the fruit [5]. Mostly, the *C. papaya* plant is propagated by seeds, but this method of propagation forms dissimilarity between the seedlings and the mother plant and limits seed-based propagation of *C. papaya* in commercial quantities [6,7]. Therefore, clonal multiplication by utilizing somatic embryogenesis was adopted to overcome this problem [8]. Somatic embryogenesis is a process of generating embryogenic cells from somatic cells after exposure to induction conditions [9]. Somatic embryogenesis can be used to increase plant production through the propagation of elite cultivars that produce fruits with high economic value [10]. There are many reports on somatic embryogenesis of *C. papaya* using immature zygotic embryos (IZE) [10,11,12,13,14]. However, all methods described in those reports were unsuitable for propagation of plantlets on an economic scale because of high incidence of abnormalities among the regenerated embryos and callus formation at the radical end of somatic embryos (SEs), which prevents conversion of SEs to plantlets [14].

Different *C. papaya* cultivars respond differently to in vitro cultures. The *C. papaya* “Taiping” produces five to six times more shoots in cultures compared to the *C. papaya* “Eksotika”, even when the medium is controlled for. Also, the continuous usage of papaya cultures to generate shoots in liquid media produced abnormal shoots. The *C. papaya* “Eksotika” was more susceptible to abnormality than the *C. papaya* “Taiping” [15]. Callus induction for somatic embryogenesis is affected by *C. papaya* cultivars. For instance, the *C. papaya* “Solo” produced more calluses than the explants from the *C. papaya* “Sunrise” in the same type of media [16]. The different cultivars of *C. papaya* responded differently during embryogenesis that took place in the same concentrations of 2,4-Dichlorophenoxyacetic acid (2,4-D) [11].

Phloroglucinol (PG) is not familiar to many researchers in plant biotechnology labs and is mostly used as a complement in combination with other plant growth regulators, as its actual effect is hidden by commonly used plant growth regulators [17]. Phloroglucinol (1,3,5-trihydroxybenzene) has growth-promoting properties. PG gave a positive effect and enhanced the germination of *Feijoa sellowiana* that was regenerated through somatic embryogenesis when used in induction media [18]. Many researchers reported the significant effect of PG in stimulating shoot development when added to culture media [19,20], enhancing root induction in *Bacopa monnieri* [21], enhancing root induction in *Rosa damascena* [22], enhancing rooting frequency in *Asparagus racemosus* [23], enhancing micro-shoot rooting and plant survival of walnut trees during acclimatization [24], and enhancing bud induction responses and formation in *Capsicum annuum* on the inverted hypocotyls [25]. The presence of PG in culture medium of nodal segments from *Stevia rebaudiana* reduced the time needed for rooting and acclimatization. It also improved the photosynthetic activity in new leaves grown ex vitro and the survival rate of plants [26].

**Figure 1:**
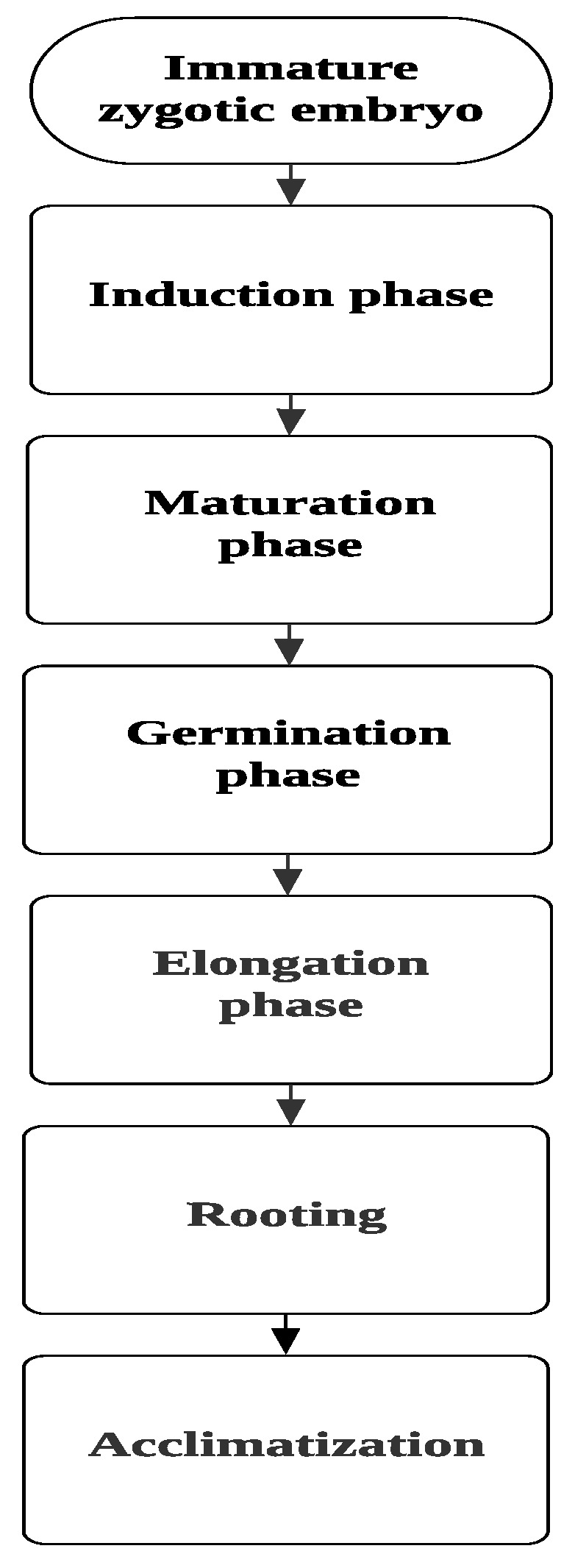
General phases in somatic embryogenesis process.

This research aimed to create a suitable and highly efficient protocol for regeneration of SEs in *C. papaya* from IZE. For that, different media with different plant growth regulator concentrations were examined to standardize the different developmental stages during somatic embryogenesis steps, starting from the induction stage all the way to the acclimatization stage (Figure 1).

## 2. Results and Discussion

### 2.1. Induction Phase

**Figure 2:**
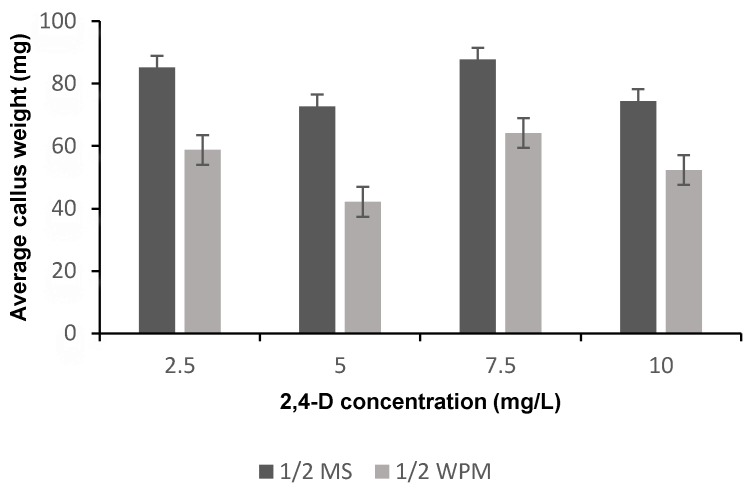
The means of average callus weight (g) on different media supplemented with different 2,4-D concentrations after 6 weeks of culture. MS: Murashige and Skoog; WPM: McCown Woody Plant.

**Figure 3:**
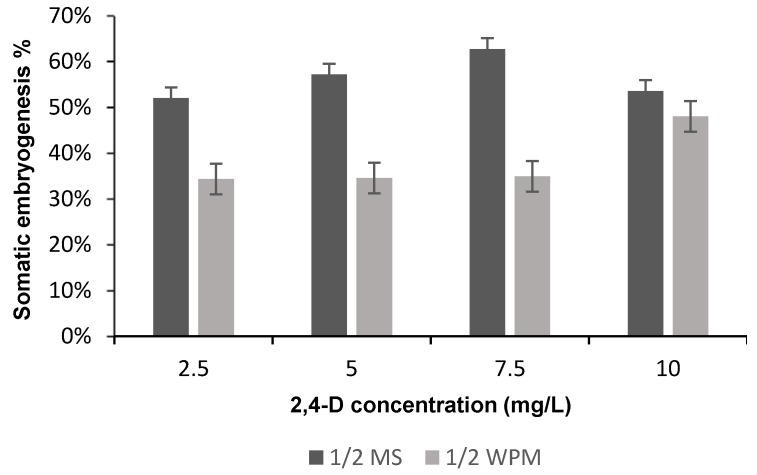
The means of the somatic embryogenesis percentage on different media supplemented with different 2,4-D concentrations after 6 weeks of subculture.

After 3 weeks of culturing, most IZE produced calluses on media containing 2,4-D but not in the control medium (i.e., medium without 2,4-D). After 6 weeks of culturing, the majority of the Petri dishes showed friable yellow calluses with SEs formation. The average callus weight and percentage of somatic embryogenesis were affected significantly (*p* < 0.05) with media composition (Table 1), whereas the average callus weight and percentage of somatic embryogenesis were affected non-significantly (*p* < 0.05) with the 2,4-D concentration and the interaction between media and 2,4-D concentration (Table 1). The highest average callus weight and somatic embryogenesis percentage were recorded in IM3 treatment [half strength Murashige and Skoog (½ MS) supplemented with 7.5 mg L^−1^ 2,4-D] (Figure 2 and Figure 3). The highest somatic embryogenesis percentages in half strength McCown Woody Plant (½ WPM) were recorded in IM8 treatment (½ WPM supplemented with 10 mg L^−1^ 2,4-D) (Figure 3).

Somatic embryogenesis is one of the pathways for in vitro plant regeneration. The relationship between culture medium composition and explant type leads to somatic embryo formation, but this relationship is complex and remains poorly understood. Most researchers use the classical approach of manipulating the ratios of plant growth regulators to optimize the quality and the number of embryos. In spite of this, several species and varieties do not respond to this classical approach and need additional optimization through the manipulation of other chemicals or physical factors [27]. A complete understanding of the factors that regulate induction, maturation, and germination of SEs will increase the efficiency of protocols for *C. papaya* propagation. Callus induction in *C. papaya* is frequently accomplished by adding 2,4-D to MS or ½ MS medium [11,28,29,30]. In general, small consideration has been offered to the constituents of media used for *C. papaya* propagation by somatic embryogenesis.

In our study, two different types of media were tested in combination with several concentrations of 2,4-D to induce embryogenic callus formation. The types of media differed in their nitrogen level, total ionic strength, ammonium-to-nitrate ratio, and ammonium concentration. It may be inferred that one of these nutritional parameters could be responsible for the differences in embryogenic response.

The best 2,4-D concentration for induction of somatic embryogenesis in ½ MS medium was 7.5 mg L^−1^, but the differences among treatment levels were not statistically significant. Our results differed from those of Fitch and Manshardt [11], who found that ½ MS medium augmented with 5 mg L^−1^ 2,4-D gave the best result for induction somatic embryogenesis. If this difference is real, it may be due to the differences in *C. papaya* cultivars.

### 2.2. Maturation Phase

**Figure 4:**
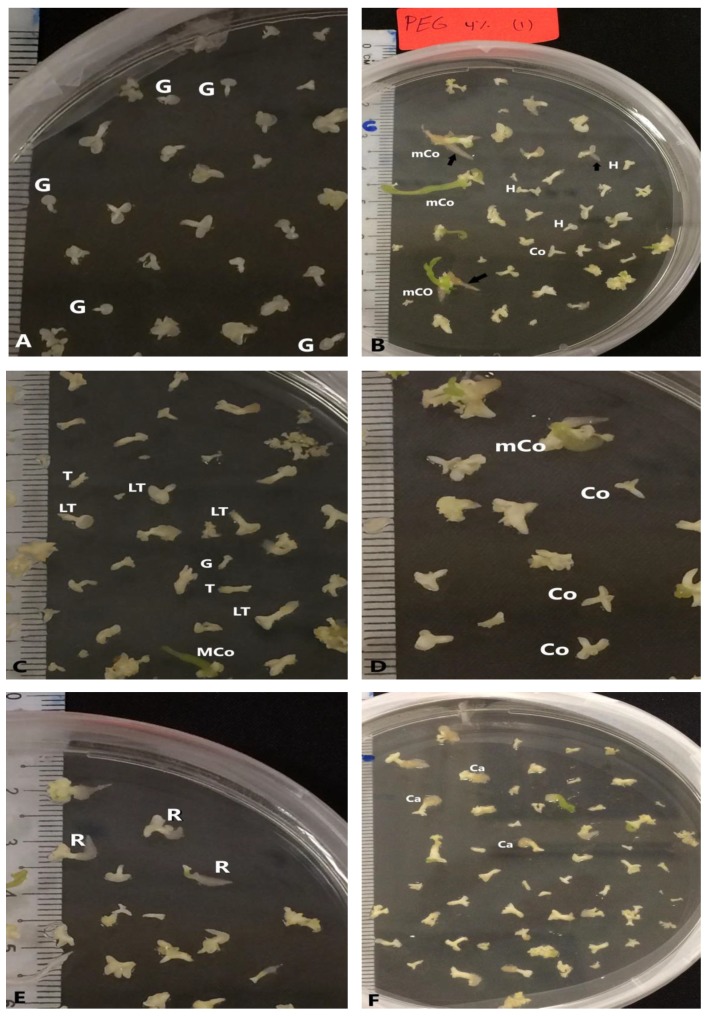
Different developmental stages of somatic embryos during maturation phase: (**A**) globular shape (G) of somatic embryo, (**B**) heart shape (H) and mature cotyledon stage (MCo) of somatic embryo, (**C**) torpedo stage (T), late torpedo stage (LT) and mature cotyledon stage (MCo) of somatic embryos, (**D**) cotyledon stage (Co) and mature cotyledon stage (MCo) of somatic embryos, (**E**) somatic embryos with root (R), (**F**) somatic embryos with callus (Ca) at the base end.

**Figure 5:**
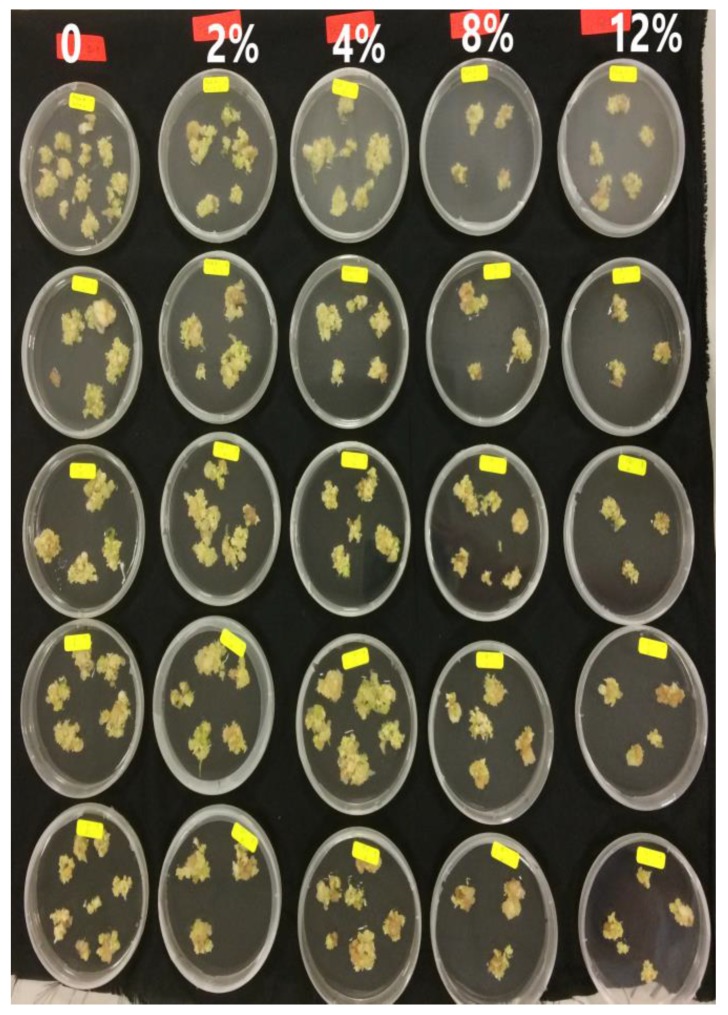
Embryogenic callus after 6 weeks on maturation media supplemented with different concentrations of polyethylene glycol 8000 (PEG) (0, 2, 4, 8, 12%) (M1–M5).

The potential for somatic embryo development in response to the various maturation treatments was analyzed through the mean number of SEs in each stage (Figure 4). The embryogenic callus induced in IM1 medium (½ MS supplemented with 2.5 mg L^−1^ 2,4-D) was used to examine the effect of polyethylene glycol 8000 (PEG) on the maturation of SEs. The number of large SEs (larger than 3 mm) and the early torpedo shape were affected significantly (*p* < 0.05) with the PEG concentration (Table 2 and Figure 5).

Numerous studies demonstrated positive effects of PEG on somatic embryo maturation [31,32] and germination [31,33]. The quality of SEs can be improved by using PEG to decrease the osmotic potential in the maturation medium [34]. PEG cannot infiltrate into the plant cells, thus it limits water absorbance by creating water stress under in vitro conditions and simulates drought stress during SEs development [33,35]. Water stress changes the DNA methylation pattern and causes changes in expression of genes encoding proteins crucial for somatic embryo development [36]. Other researchers found that the PEG concentration in media had a positive correlation with the level of endogenous free proline [37]. The results of the present study are similar to those of Langhansova et al. (2004), who showed that PEG enhanced root meristem organization of torpedo-stage embryos during somatic embryogenesis in *Panax ginseng*. M5 treatment was chosen for further experiments because it increased the number of cotyledonary embryos during the maturation phase.

**Figure 6:**
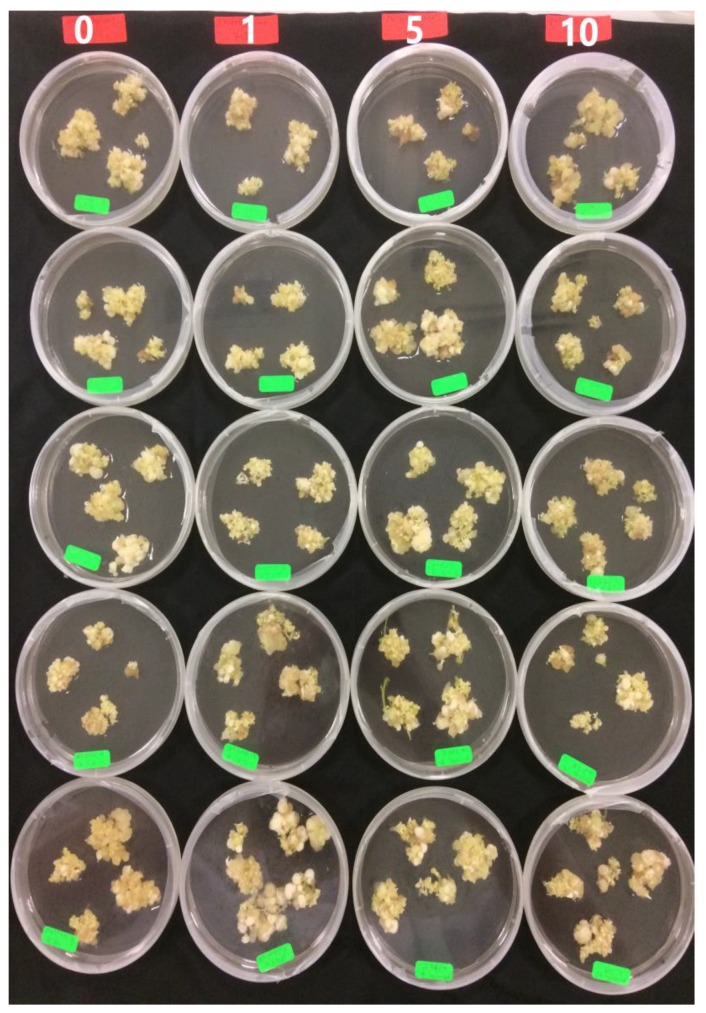
Embryogenic callus after 6 weeks on maturation media supplemented with different concentrations of phloroglucinol (PG) (0, 1, 5, 10 mg L^−1^) (M6–M9).

The embryogenic callus induced in IM3 medium (½ MS supplemented with 7.5 mg L^−1^ 2,4D) was used to examine the effect of PG on the maturation of SEs. The number of cotyledon shape embryos was affected significantly (*p* < 0.05) with PG concentration (Table 3 and Figure 6). PG enhanced the germination of *Feijoa sellowiana* regenerated through somatic embryogenesis when used in the induction media [18]. PG was necessary during maturation and plant development during somatic embryogenesis of petal explants from *Rosa hybrida* L. “Arizona” [38]. It also played a critical role in controlling hyperhydricity throughout the lignification process in *Acca sellowiana* [39]. Until now, there has not been any published research on the effect of PG on the maturation phase of SEs of *Carica papaya* “Eksotika”.

M8 treatment was chosen for further experiments because it increased the number of cotyledonary embryos during the maturation phase. The positive results of PG in maturation medium of the present study are similar to those of Murali et al. [38], who showed that PG enhanced the germination of SEs in *Rosa hybrida* L. “Arizona”. The positive effect might be explained by a decrease in the amount of phenolic compounds in explants cultured in media augmented with PG [18]. Other researchers have stated that the addition of PG to in vitro culture medium acts as a synergist with auxin and stimulates shoot development [20]. The presence of PG in the culture media enhanced the production of various metabolites and enzymes (e.g., catalase and peroxidase, which are the oxidative enzymes) in *Aristolochia tagala* callus that originated from leaves. PG also increased the production of protein and carbohydrate (metabolites). In addition, it decreased the amount of polyphenol oxidase [40].

### 2.3. Germination Phase

**Figure 7:**
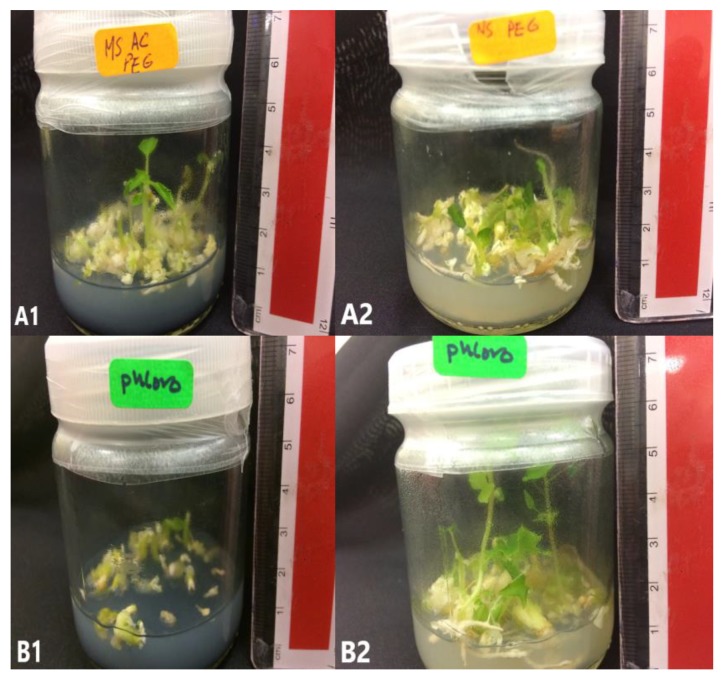
*C. papaya* “Eksotika” in germination media A1, explant germinated in G1 media. A2, explant germinated in G2 media. B1, explant germinated in G3 media. B2, explant germinated in G4 media.

To study the effect of activated charcoal (AC) during the germination phase, SEs grown in M5 and M8 were used as source of explant. The germination percentage of SEs was not affected with AC and source of explant (Table 4 and Figure 7).

Many researchers have demonstrated positive effects of AC in plant tissue cultures, especially in supporting growth and development of plant tissues [41,42]. AC helps in the induction [43] and the maturation [44] of SEs. The addition of AC to culture media may either encourage or restrain growth, depending on the species and the tissues used [45]. The present study demonstrated nonsignificant effects of AC during somatic embryo germination of *C. papaya*, regardless of the source of SEs, which came from M5 or M8.

### 2.4. Elongation Phase

**Figure 8:**
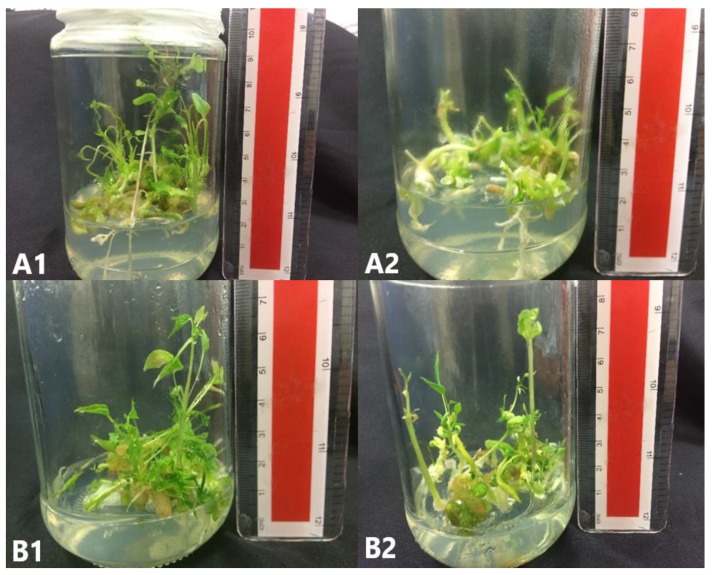
*C. papaya* “Eksotika” in elongation media **A1** and **A2**, explant germinated in EL1 media. **B1** and **B2**, explant germinated in EL2 media.

**Figure 9:**
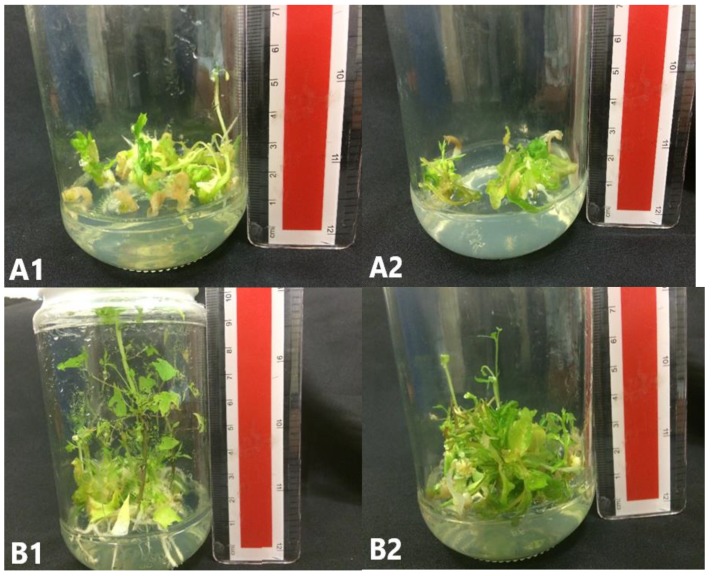
*C. papaya* “Eksotika” in elongation media **A1** and **A2**, explant germinated in EL3 media. **B1** and **B2**, explant germinated in EL4 media.

The percentage of somatic embryo survival was recorded 6 weeks after culture in Elongation media (EL) medium (Table 5). A single medium was used for elongation of SEs. The source of SEs (which came from germination medium supported with AC or not) did not significantly affect (*p* < 0.05) the survival percentage after the elongation phase (Figure 8 and Figure 9).

### 2.5. Rooting Phase

Root induction and development is a critical parameter for in vitro plant regeneration in economic scales. The major problem faced in developing transgenic *C. papaya* plants is low rooting efficiency of regenerated shoots [13]. Many factors influence the development of roots in in vitro cultures, such as auxin type and concentration, shoot quality, donor age, and temperature [46]. The interaction of phytohormone during root development and growth is not fully understood [47]. Auxin plays a large role in regulating roots during growth and development [47,48]. It also regulates plant responses to the environment such as phototropism, gravitropism, and thigmotropism [49]. Indole-3-butyric acid (IBA) was reported by many researchers as the best auxin for root induction in *C. papaya* [50,51,52]. IBA is better than other plant growth regulators, such as indole-3-acetic acid (IAA), 1-Naphthaleneacetic acid (NAA), or *p*-Chlorophenoxyacetic acid (*p*CPA) for root initiation of *C. papaya*. IBA has a greater ability to promote rooting with less callus formation compared with other types of auxin [52]. In the present study, we tested different additives (PG and riboflavin) that may promote rooting. PG enhanced root induction in *Jatropha curcas* L. in the presence of IBA [53], *Prunus avium* [54], and apple cultivars [55].

**Figure 10:**
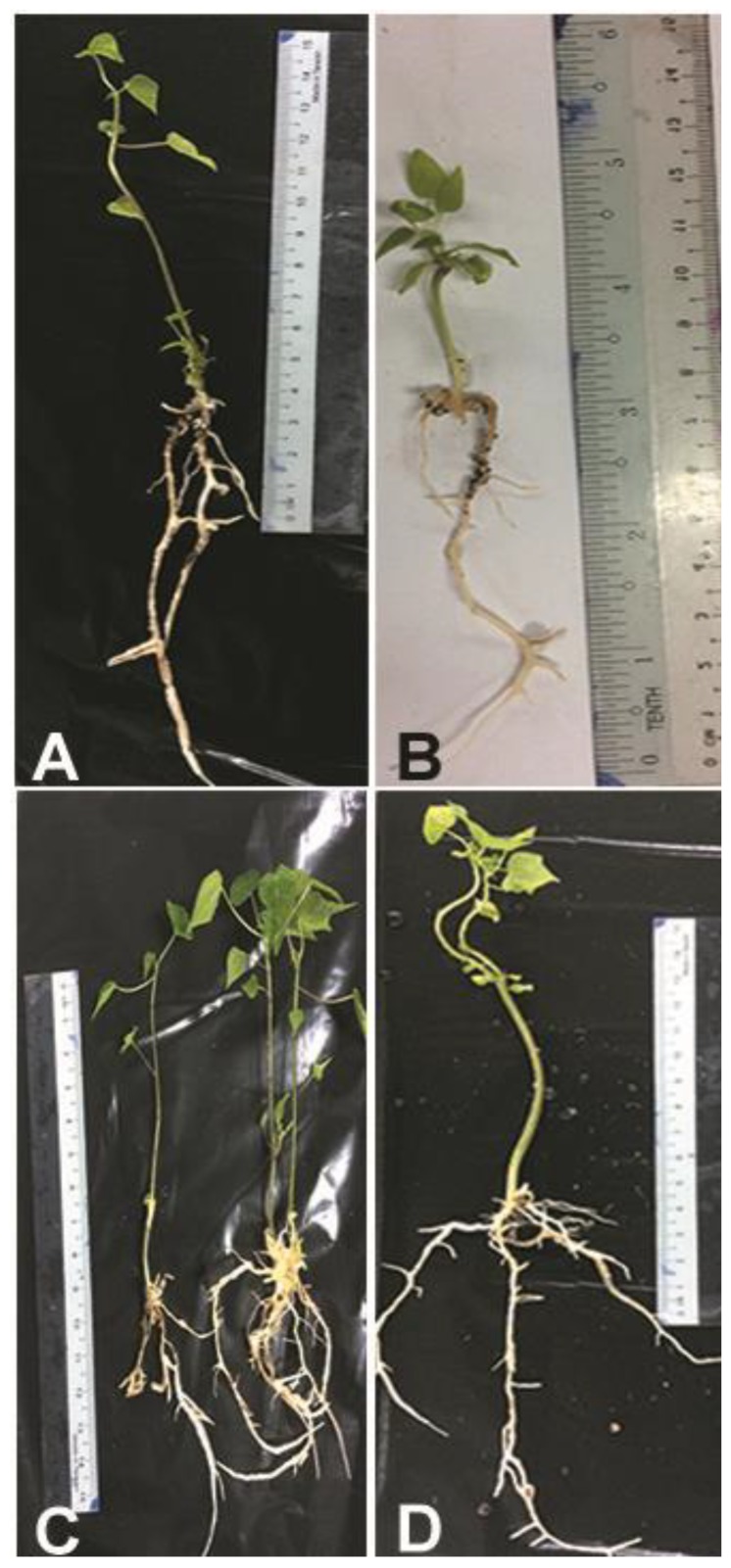
*C. papaya* “Eksotika” (**A**) explants rooted in R1 medium, (**B**) explants rooted in R2 medium, (**C**) explants rooted in R3 medium, (**D**) explants rooted in R4 medium.

Four types of media were used in rooting experiments to study the effects of riboflavin and PG on root formation. The survival percentage of SEs was affected significantly (*p* < 0.05) with the presence of riboflavin. The largest survival percentage was recorded in R2 media. The survival rate of SEs was affected significantly (*p* < 0.05) with presence of PG (Table 6 and Figure 10).

Riboflavin is produced by plants and used as a catalyst in diverse metabolic pathways. The role of riboflavin in improving root induction and development has been registered in many studies [52,56,57]. Adventitious root systems for papaya (*Carica papaya* L.) were enhanced by exposure of shoots to a medium containing IBA for 2 days before transfer to a hormone-free medium containing riboflavin to enhance shoot and root formation by increasing the shoot length and decreasing callus formation at the base end of the stem [52]. Root formation on apple (Malus) shoots cultured in vitro was increased after an incubation in the dark medium supplemented with IBA and riboflavin. Removing riboflavin significantly affected the number of roots formed [56]

In the present study, the survival percentage of explant affected significantly (*p* < 0.05) with the presence of riboflavin, where added riboflavin to root media increased the survival percentage of explant. Our result agrees with those of Drew, et al. [58], who found that riboflavin promoted root initiation.

In the present study, the length of roots was affected significantly (*p* < 0.05) with the presence of PG. Our results are similar to those of many previous studies demonstrating a positive role of PG for the enhancement of root frequency [23] and root induction [21,59] in different plant species.

### 2.6. Acclimatization

**Figure 11:**
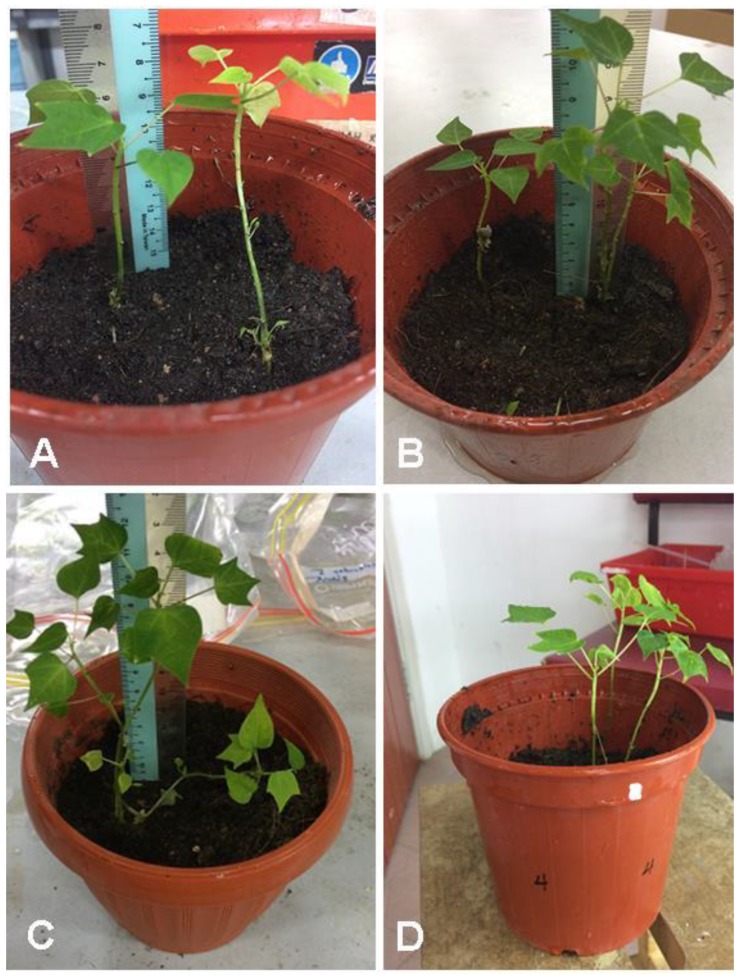
*C. papaya* “Eksotika” after one month: (**A**) explants regenerated from R1 medium, (**B**) explants regenerated from R2 medium, (**C**) explants regenerated from R3 medium, (**D**) explants regenerated from R4 medium.

The SEs rooted in different rooting media were transferred to peat moss soil for acclimatization, and the survival percentages were recorded after 1 month. The percentage of survival after acclimatization phase root media was affected significantly (*p* < 0.05) with root media (Table 7 and Figure 11).

Peat moss soil was used for acclimatization. The highest survival percentage was observed with germinated explant grown in R4 medium and transferred to peat moss soil. Our results are similar to those of many previous studies demonstrating a positive role of PG for the enhancement of rooting and plant survival of walnut trees during acclimatization [24].

## 3. Materials and Methods

### 3.1. Fruit Collection and Sterilization

Fruits of *C. papaya* cv. “Eksotika” were collected from organic farms near Desaru Bandar Penawar, Johor Bahru, which is 391 km southwest of Kuala Lumpur. The fruits were 95–100 days old when harvested. The fruits were sprayed with 70% ethanol and saved in an insulated icebox (4–6 °C) for 8 h until they were brought to the laboratory. The fruits were sprayed again with 70% ethanol, then washed with soap under running faucet water for approximately 15 min. The “Eksotika” fruits were sprayed three times with 70% ethanol in a “horizontal laminar flow hood” and cut open using a sterile sharp knife. The white creamy seeds were detached using a sterile spoon, and IZE was isolated from white creamy seeds by using forceps and a sterile dissecting blade.

#### 3.1.1. Culture Conditions

All media in this paper were adjusted to pH 5.8 before adding gelrite or agar and were sterilized by autoclaving at 104 kPa (121 °C) for 20 min. All cultures in the induction and the maturation phases were maintained at 27 ± 1 °C in a culture room under dark conditions. For germination, elongation, and rooting stages, the cultures were maintained in a culture room at 27 ± 1 °C under a 16/8 h light/dark condition with 45 ± 5 μmol m^−2^ sec^−1^ light intensity from cool-white fluorescent tubes (Phillips, 32W 48in T8). All chemicals used in this paper were bought from Sigma-Aldrich except gelrite, sucrose, Murashige and Skoog (MS) media [60], and McCown Woody Plant (WPM) media [61], which were bought from Duchefa.

#### 3.1.2. Induction Phase

In the induction phase, eight treatments were used for induction of the embryogenic callus. Each treatment consisted of one type of medium with one concentration of 2,4-D (Table 8).

Induction of embryonic callus was performed in Petri dishes (90 mm × 25mm) including 20 mL of medium. Ten Petri dishes were used for each treatment with nine IZE in each plate. Subculturing was done biweekly for 6 weeks. Average callus fresh weight and somatic embryogenesis percentage were recorded biweekly as follows:

$$Average\;callus\;fresh\;weight=\frac{Weight\;of\;all\;embryogenc\;callus\;in\;one\;plate}{Total\;number\;of\;IZE}$$

where the weight of the induced callus was measured by using balance under a laminar flow hood condition.

$$Somatic\;embryogenesis\;\%=\frac{Number\;of\;IZE\;showes\;somatic\;embryos\;}{Totalnumber\;of\;IZE}\;x100\%$$

### 3.2. Maturation Phase

The embryogenic calli that showed the best percentage of somatic embryogenesis induction in different induction media (IM1 and IM3) were used to examine nine maturation treatments with different concentrations of PEG or PG (Table 9).

The 2,4-D concentration in each maturation treatment was initially half of that in the corresponding induction treatment (i.e., for the first subculture, M1–M5 contained 1.25 mg L^−1^, and M6–M9 contained 3.75 mg L^−1^). These 2,4-D amounts were halved again for the second subculture (i.e., M1–M5: 0.63 mg L^−1^ and M6-M9: 1.88 mg L^−1^), and the third subculture was done without 2,4-D. Five Petri dishes (90 mm × 25 mm) were used for each treatment, and 0.4 g of embryogenic callus was transferred to each Petri dish. Subculturing was done biweekly for 8 weeks. The size of SEs (3 mm > small, 3 mm ≤ large) and the numbers of SEs at various developmental stages (globular, heart, cup-shape, torpedo, and cotyledon shape) were recorded after 8 weeks.

### 3.3. Germination Phase

The SEs that showed the best growth in different maturation treatment, M5 and M8, were used to test germination treatments. Four germination treatments with or without AC were used to examine the germination phase (Table 10). Five glass jars (30 mL medium in each) were used for each treatment. Each jar included 25 cotyledonary-stage embryos (length >3 mm). After 6 weeks of culturing, the survival rate (expressed as a percentage) was evaluated as follows:

$$Survival\;rate=\frac{Number\;of\;survival\;somatic\;embryos\;after\;germination\;phase}{Total\;number\;of\;somatic\;embryos\;\left(25\right)}\;x100\%$$

#### Elongation Phase

Explants from all germination treatments were used to examine the elongation treatments (Table 11). Five glass jars (50 mL of medium in each) with five SEs were utilized for each treatment. After 6 weeks of culturing, the survival rate (expressed as a percentage) was evaluated.

$$Survival\;rate=\frac{Number\;of\;survival\;somatic\;embryos\;after\;elongation\;phase}{Total\;number\;of\;somatic\;embryos\;\left(5\right)}\;x100\%$$

### 3.4. Rooting Phase

For the rooting phase, the explants grown in EL2 and EL4 treatments were used (Table 12) to study the effect of riboflavin and PG on rooting. After 6 weeks of culturing, the length and the number of roots formed as well as the callus formation on the roots were recorded.

### 3.5. Acclimatization

After the rooting phase, in vitro plantlets from all root treatments were transferred to plastic pots (8 × 6 cm) filled with peat moss soil. The vermiculite and the agar medium were gently rinsed off the roots by using autoclaved distilled water before planting. After that, the potted plantlets covered with a transparent plastic bag were kept in a culture room under the same conditions described in the germination phase for 3 weeks and were watered every 3 days.

### 3.6. Statistical Analysis

In this research, the whole experiments were set up in a “completely randomized design”. All quantitative data mentioned as percentages were firstly examined for normal distribution. All the variables in different phases of somatic embryogenesis were evaluated with one-way or two-way analysis of variance (ANOVA). The differences between means were scored with the “Duncan multiple range test” using “IBM SPSS Statistics 20 software”.

## 4. Conclusions

An efficient protocol for regeneration of *Carica papaya* L. cv. “Eksotika” SEs from IZE was developed. The percentage of somatic embryogenesis was affected significantly (*p* < 0.05) with media composition, whereas the percentage of somatic embryogenesis was affected non-significantly (*p* < 0.05) with the 2,4-D concentrations and the interaction between media and 2,4-D concentrations. During the maturation phase, the number of large SEs (larger than 3 mm) and the early torpedo shape were affected significantly (*p* < 0.05) with the PEG concentrations, while the number of cotyledon shape embryos was affected significantly with the PG concentrations. The germination percentage of SEs during the germination phase was affected non-significantly (*p* < 0.05) with the presence AC and the source of explant. The survival percentage of explants during the rooting phase was affected significantly (*p* < 0.05) with the presence of riboflavin. For acclimatization, the percentage of survival after the acclimatization phase was affected significantly (*p* < 0.05) with the root media.

## Figures and Tables

**Table 1:** Analysis of variance of the effect of media, 2,4-D concentrations and interaction between media and 2,4-D concentration on the average callus weight and percentage of somatic embryogenesis for induction of somatic embryogenesis in Eksotika.

	Average Callus Weight		Percentage of Somatic Embryogenesis	
	F	*p*-Value	F	*p*-Value
Media (M)	20.708	0.000	11.595	0.001
2,4-D Concentration (C)	2.186	0.102	0.385	0.765
M * C	0.108	0.955	0.774	0.514

**Table 2:** Comparison of embryo size classes, somatic embryo (SE) developmental stage, number of SEs with callus formation at the base, and number of SEs that formed roots in M1–M5 media after 8 weeks of subculture. All values are means ± SD.

Media	Large Embryos	Small Embryos	Somatic Embryo Developmental Stage					Callus	Root
			Globular	Heart	Torpedo	Early Torpedo	Cotyledonary		
**M1**	20.80 ± 4.266c	27.20 ± 4.63a	15.20 ± 2.25a	5.20 ± 3.56b	18.20 ± 5.22a	6.40 ± 4.28b	6.20 ± 1.64c	3.6 ± 3.78a	6.40 ± 1.151b
**M2**	30.40 ± 1.871b	19.60 ± 3.72a	11.00 ± 4.03a	5.00 ± 2.55b	18.80 ± 3.77a	6.20 ± 3.42b	13.60 ± 7.23ab	1.00 ± 0.71ab	12.20 ± 2.77ab
**M3**	31.00 ± 5.339b	17.80 ± 4.28a	11.00 ± 3.84a	7.00 ± 3.61ab	13.60 ± 5.94a	4.20 ± 2.59b	16.40 ± 5.60a	1.00 ± 1.23ab	13.60 ± 2.89ab
**M4**	37.00 ± 7.810b	26.00 ± 2.37a	9.60 ± 1.28a	8.80 ± 3.97ab	17.60 ± 1.67a	13.80 ± 7.36a	17.60 ± 8.02a	1.20 ± 1.10ab	10.20 ± 1.88ab
**M5**	48.80 ± 5.111a	21.80 ± 6.96a	14.00 ± 2.60a	10.00 ± 1.23a	20.00 ± 7.94a	16.40 ± 5.51a	17.00 ± 8.00a	0.00 ± 0.00b	18.60 ± 5.26a
***p*** **-value**	0.00	0.323	0.261	0.080	0.415	0.003	0.069	0.071	0.068
**F-value**	12.612	1.248	1.428	2.439	1.033	5.912	2.580	2.554	2.591

Means followed by the same letter in same column are not significantly different at *p* < 0.05 according to the Duncan multiple range test.

**Table 3:** Comparison of embryo size classes, somatic embryo developmental stage, number of SEs with callus formation at the base, and number of SEs that formed roots in M6-M9 medium after 8 weeks of subculture. All values are means ± SD.

Media	Large Embryos	Small Embryos	Somatic Embryo Developmental Stage					Callus	Root
			Globular	Heart	Torpedo	Early Torpedo	Cotyledonary		
**M6**	27.00 ± 5.24a	25.20 ± 4.22a	13.60 ± 1.48a	10.00 ± 2.12a	14.80 ± 2.28a	10.60 ± 5.94a	7.20 ± 1.79b	6.20 ± 3.49a	5.60 ± 2.07a
**M7**	30.00 ± 1.87a	26.60 ± 3.09a	10.80 ± 2.77a	10.60 ± 2.88a	19.80 ± 2.68a	11.60 ± 1.14a	8.00 ± 3.67b	3.00 ± 1.23a	9.60 ± 5.03a
**M8**	32.80 ± 7.50a	41.20 ± 11.44a	26.20 ± 9.91a	10.20 ± 4.21a	15.00 ± 8.75a	9.20 ± 2.28a	13.20 ± 4.38a	3.80 ± 2.17a	9.80 ± 3.42a
**M9**	31.80 ± 7.79a	27.60 ± 5.51a	15.80 ± 2.68a	5.60 ± 2.41b	16.60 ± 5.86a	11.20 ± 4.50a	10.00 ± 2.35ab	4.20 ± 4.15a	12.20 ± 7.33a
***p*** **-value**	0.474	0.262	0.161	0.060	0.478	0.917	0.042	0.401	0.433
**F-value**	0.876	1.461	1.957	3.034	0.867	0.167	3.452	1.041	0.965

Means followed by the same letter in same column are not significantly different at *p* < 0.05 according to the Duncan multiple range test.

**Table 4:** Comparison of means germination percentage after 6 weeks of germination. All values are means ± SD.

Media	Mean ± SD	F Value	*p*-Value
**G1**	53.60 ± 4.60	3.881	0.084
**G2**	42.40 ± 4.38		
**G3**	56.80 ± 5.90	2.113	0.184
**G4**	46.40 ± 5.40		
***p-*** **value**	0.144		
**F-value**	2.07		

**Table 5:** Comparison of means survival percentage after 6 weeks in elongation media. All values are means ± SD.

Media	Mean ± SD	F Value	*p*-Value
**EL1**	43.37 ± 6.13a	2.134	0.182
**EL2**	55.95 ± 7.43a		
**EL3**	44.09 ± 3.48a	3.405	0.102
**EL4**	57.80 ± 7.04a		
***p*** **-value**	0.188		
**F-value**	1.80		

Means followed by the same letter are not significantly different at *p*<0.05 according to the Duncan multiple range test.

**Table 6:** Comparison of survival (%), root number, root length (cm), shoot length (cm), leaf number, and callus formation (%) at the base of SEs after 6 weeks of culture in different rooting media. All values are means ± SD.

SEs Source	Medium Type	Survival (%)	Root Number		Root Length	Shoot length	Leaf Number	Callus Formation	
EL2	R1	52.75 ± 5.75b		2.25 ± 0.98a	6.25 ± 2.63ab	7.00 ± 2.45a	4.25 ± 0.50a	0.00 ± 0.00a	
EL2	R2	93.75 ± 4.16a		2.50 ± 1.29a	3.50 ± 1.73b	4.75 ± 1.26a	3.00 ± 1.41a	14.50 ± 4.26a	
EL4	R3	83.5 ± 4.76a		3.00 ± 1.16a	10.25 ± 3.86a	8.00 ± 2.16a	4.50 ± 1.29a	29.00 ± 3.41a	
EL4	R4	88.75 ± 4.00a		3.75 ± 0.50a	4.50 ± 1.29b	7.00 ± 0.82a	4.00 ± 0.82a	0.00 ± 0.00a	
Riboflavin									
F-value		14.816		0.097	3.050	2.670	2.778	2.890	
*p*-value		0.008		0.766	0.131	0.153	0.147	0.140	
PG									
F- value		0.178		1.421	7.975	0.750	0.429	2.890	
*p*-value		0.688		0.278	0.030	0.420	0.537	0.140	
Riboflavin X PG									
F-value		6.287		1.84	3.194	0.885	0.652	1.606	
*p*-value		0.004		0.174	0.044	0.497	0.635	0.224	

Means followed by the same letter are not significantly different at *p* < 0.05 according to the Duncan multiple range test.

**Table 7:** Comparison of the average acclimatization (% ± SD) of germinated SEs after 6 weeks of growth in peat moss soil.

Explant Source	Acclimatization (%)	F-Value	*p-*Value
R1 medium	55.11 ± 4.81c	0.160	0.710
R2 medium	66.67 ± 6.29bc		
R3 medium	83.33 ± 4.81ab	1.00	0.374
R4 medium	100.0 ± 00.00a		
F value	407.833		
*p*-value	0.000		

Means followed by the same letter are not significantly different at *p* < 0.05 according to the Duncan multiple range test.

**Table 8:** The treatments composition during the induction phase.

Code of Treatment Media	Composition Solid Media Treatment (Per Litter Media)	
	Media	2,4-D (mg L^−1^)
**IM1**	½ MS	2.5
**IM2**	½ MS	5
**IM3**	½ MS	7.5
**IM4**	½ MS	10
**IM5**	½ WPM	2.5
**IM6**	½ WPM	5
**IM7**	½ WPM	7.5
**IM8**	½ WPM	10

All treatments were supplemented with 100 mg L^−1^ L-glutamine, 50 mg L^−1^ *myo*-inositol, 45 mg L^−1^ adenine sulphate, 0.33% gelrite, and 6% sucrose.

**Table 9:** The treatments composition during the maturation phase.

Source of SEs	Code of Treatment Media	Composition Solid Media Treatment (Per Litter Media)		
		Media	PEG (%)	PG (mg L^−1^)
**IM1**	M 1	½ MS	0	0
**IM1**	M 2	½ MS	2	0
**IM1**	M 3	½ MS	4	0
**IM1**	M 4	½ MS	8	0
**IM1**	M 5	½ MS	12	0
**IM 3**	M 6	½ MS	0	0
**IM 3**	M 7	½ MS	0	1
**IM 3**	M 8	½ MS	0	5
**IM 3**	M 9	½ MS	0	10

All treatments were supplemented with 100 mg L^−1^ L-glutamine, 100 mg L^−1^ *myo*-inositol, 68 mg L^−1^ adenine sulphate, 0.38% gelrite, and 3% sucrose.

**Table 10:** The treatments composition during the germination phase.

Source of SEs	Code of Treatment Media	Composition Solid Media Treatment (Per Litter Media)	
		Media	AC (0.05%)
**M5**	G 1	½ MS	√
**M5**	G 2	½ MS	0
**M8**	G 3	½ MS	√
**M8**	G 4	½ MS	0

All germination media supplemented with 3% sucrose and 0.8% agar. √: presence of activated charcoal; 0: absence of activated charcoal (AC).

**Table 11:** The treatments composition during elongation phase.

Source of SEs	Code of Treatment Media	Media
**G1**	EL 1	½ MS
**G2**	EL 2	½ MS
**G3**	EL 3	½ MS
**G4**	EL 4	½ MS

All treatments were supplemented with 1 mg L^−1^ gibberellic acid (GA3), 0.5 mg L^−1^ indole-3-butyric acid (IBA), 100 mg L^−1^ *myo*-inositol, and 3.76 mg L^−1^ riboflavin.

**Table 12:** The composition of treatment media during the rooting phase.

Source of SEs	Code of Treatment Media	Composition Solid Media Treatment (Per Litter Media)	
		Riboflavin 3.76 mg L^−1^	PG 7.9 mg L^−1^
**EL2**	R 1	0	0
**EL2**	R 2	√	0
**EL4**	R 3	0	0
**EL4**	R 4	0	√

R1–R4 treated for 4 days in MS medium augmented with 2 mg L^−1^ indole-3-butyric acid (IBA), 3% sucrose, and 0.38% gelrite, then transferred to four types of media supported by vermiculite and 3% sucrose.
